# Standing NanoNeedle Arthroscopy of the Distal Interphalangeal Joint for Removal of Osteochondral Fragments of Distal P2 and the Extensor Process in a Horse

**DOI:** 10.3390/ani16081168

**Published:** 2026-04-10

**Authors:** Nicole A. I. Phillips, Lisa A. Fortier, Christina S. Cable, Aimee C. Colbath

**Affiliations:** 1 Department of Clinical Sciences, Cornell University, 930 Campus Road, Ithaca, NY 14853, USA; np482@cornell.edu (N.A.I.P.); lfortier@avma.org (L.A.F.); 2 Early Winter Equine, 1420 Ridge Road, Lansing, NY 14882, USA; cscable@earlywinterequine.com

**Keywords:** arthroscopy, standing arthroscopy, osteochondral fragments, equine, Arthrex NanoNeedle

## Abstract

Background: In horses, standing arthroscopy offers a valuable alternative to general anesthesia for the reduction of anesthetic-associated risks, and provides improved visualization in joints where access is facilitated by weightbearing. These techniques are particularly advantageous for patients with increased anesthetic risk or financial limitations. To the authors’ knowledge, the removal of osteochondral fragments from the distal interphalangeal joint in a standing, sedated horse using small-diameter arthroscopic equipment has not been previously reported. Case Presentation: A 7-year-old American Quarter Horse–Thoroughbred cross presented on referral for treatment of osteochondral fragmentation in the right fore distal interphalangeal joint. A standing arthroscopic approach to the distal interphalangeal joint was performed. In a weight-bearing position, debridement of the dorsal osteochondral fragments from the second and third phalanx was successfully completed, using a 2 mm NanoNeedle scope and 3.2 mm high-flow 10- degree cannula (Arthrex^®^). At one year follow-up, the horse had returned to competition and was sound at veterinary examination. Conclusion: This case demonstrates the feasibility of standing NanoNeedle arthroscopy of the equine coffin joint with improved maneuverability, surgical ergonomics, and reduced iatrogenic injury compared to a standard rigid arthroscope. The successful execution of this technique effectively expands our collection of standing arthroscopic procedures in the equine patient.

## Simple Summary

Traditional arthroscopy of equine patients is associated with the risk and financial cost of general anesthesia. Standing arthroscopic approaches provide a valuable opportunity to improve patient safety and access to care. Standing arthroscopy may be performed for diagnostic purposes only or may include therapeutic interventions. This case report details the use of a novel arthroscope, the 2 mm NanoNeedle scope (Arthrex^®^), and a 3.2 mm high-flow 10-degree cannula (Arthrex^®^) in a standing, sedated horse for the debridement of dorsal osteochondral fragments from the second and third phalanx.

## 1. Introduction

The use of needle arthroscopic equipment for diagnostic purpose has been well described in the stifle, tarsocrural, radio-carpal, middle carpal, scapulohumeral, temporomandibular joint, bicipital bursa, and caudal cervical articular process joints of horses [1,2,3,4,5,6,7]. Standing arthroscopy offers several potential advantages. It avoids the risks associated with general anesthesia, including the systemic effects of anesthetic drugs, respiratory complications related to intubation, and the recovery period following anesthesia. Additionally, weight-bearing can increase joint space in certain joints, providing a more anatomically accurate view of the region. In many cases, standing arthroscopy may also be a more cost-effective option for the client. Expanding standing arthroscopy beyond a diagnostic tool adds additional value, and standing needle arthroscopy has been shown to allow safe and effective removal of dorsal osteochondral fragmentation in the metacarpophalangeal and metatarsophalangeal joints of horses, supporting its use as a therapeutic alternative to conventional arthroscopy under general anesthesia [8].

## 2. Case Summary

**Figure 1:**
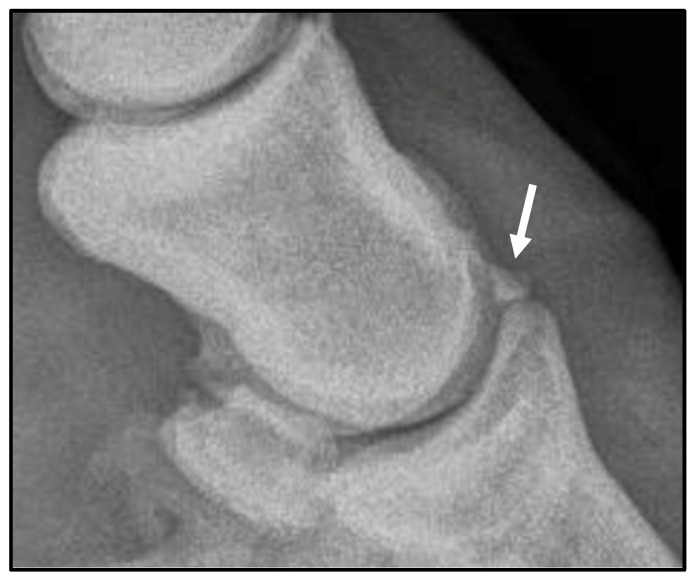
Preoperative lateral radiograph of the right fore distal interphalangeal joint, highlighting the 5 mm extensor process osteochondral fragment (arrow).

A 7-year-old American Quarter Horse–Thoroughbred cross gelding was referred to the Cornell Equine and Nemo Farm Animal Hospital for arthroscopic removal of osteochondral fragments from the right forelimb distal interphalangeal joint, previously diagnosed on field radiographs. On presentation, the gelding was bright, alert, and responsive with normal vital parameters. Musculoskeletal examination revealed mild effusion of the right coffin joint. At baseline, a Grade 3/5 right forelimb lameness (American Association of Equine Practitioners Lameness Scale) was appreciated in a straight line at the trot, and diagnostic anesthesia was not performed. On pre-operative radiographs, a 5 mm dorsal third phalanx extensor process fragment was confirmed (Figure 1).

Pre-operative medications included potassium penicillin (22,000 IU/kg IV), gentamicin (6.6 mg/kg IV), phenylbutazone (4.4 mg/kg IV), acepromazine (25 mg IV), detomidine (5 mg IV), and morphine (59 mg IV). The procedure was performed with the limb in a weight-bearing position with standing sedation using a continuous-rate infusion of detomidine hydrochloride (0.05 mcg/kg/min). A low four-point nerve block was performed aseptically with 5 mL of 2% mepivacaine hydrochloride at each site, and intra-articular anesthesia of the coffin joint was performed aseptically with 10 mL of 2% mepivacaine hydrochloride prior to the start of surgery.

The limb was clipped and aseptically prepared from the coronet band to the fetlock. A sterile glove was placed over the hoof capsule, and a sterile self-adherent cohesive bandage was wrapped over the proximal aspect of the distal limb. No additional draping was performed. The arthroscopic fluid pump (Arthrex Continuous Wave III (Model AR-6475)) was placed at the contralateral shoulder and the arthroscopy monitor was placed at ground level, lateral to the hip of the horse. This setup proved effective as the surgeon was positioned kneeling on the lateral aspect of the forelimb. A 5 mm dorsolateral incision was made to the distal interphalangeal joint using a #11 blade 3 cm proximal to the coronet band, immediately abaxial to the common digital extensor tendon. A 3.2 mm 10-degree cannula with flexible obturator was introduced to the joint, and the obturator was subsequently replaced with a 2.2 mm scope (NanoNeedle, Arthrex^®^, Naples, FL, USA). Distension of the coffin joint was maintained using an arthroscopic pump.

**Figure 2:**
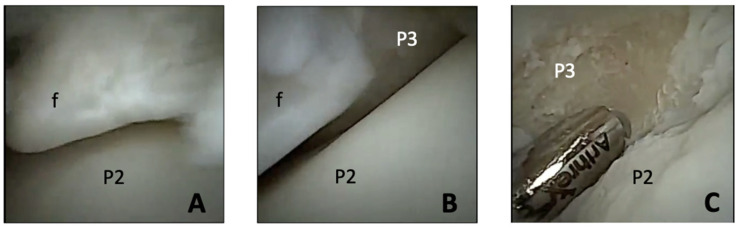
Intraoperative arthroscopic views of the distal interphalangeal joint: (**A**) extensor process fragment (f) and cartilage surface of the distal condyle of the second phalanx (P2); (**B**) border of the extensor process fragment (f), cartilage surface of the distal condyle of the second phalanx (P2), and third phalanx (P3); (**C**) debrided fracture bed of the third phalanx (P3), abnormal cartilage surface of corresponding second phalanx (P2).

**Figure 3:**
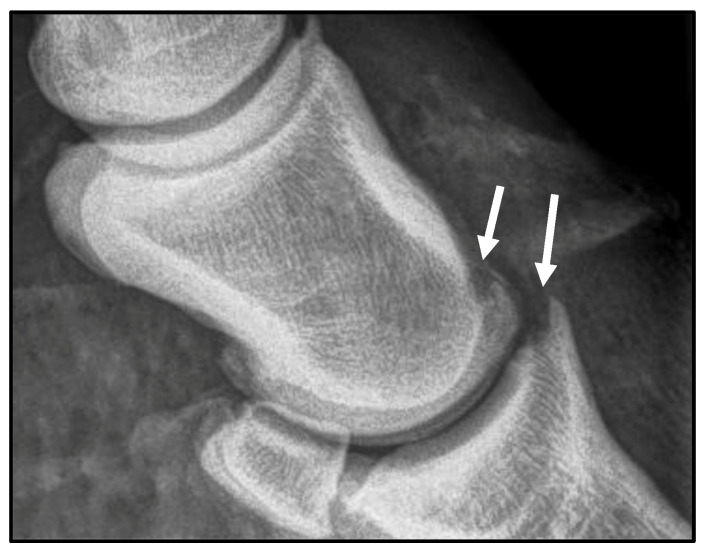
Post-operative lateral radiograph of the right fore distal interphalangeal joint, highlighting the successful removal of the osteochondral fragments from the second and third phalanx (arrows).

On initial arthroscopic exploration of the joint, mild synovitis was appreciated as well as osteochondral fragmentation of the extensor process of the third phalanx and distal, dorsal second phalanx. The distal second phalanx fragment was approximately 2 mm in diameter and not previously identified on radiographs. The remaining cartilage of the weight-bearing surfaces was in good health (Figure 2). A second arthroscopic portal was made into the dorsomedial aspect of the joint using the same technique as described above to introduce instrumentation for fragment removal and debridement. Using a combination of 4 mm Ferris–Smith rongeurs and a 2 mm Nano Oval Burr (Arthrex^®^), the fragments were successfully removed from the second and third phalanges. A size 00 curette was used to debride the fragment beds to healthy subchondral bone. Intraoperative radiography confirmed complete fragment removal (Figure 3). A total of 18 L of sterile Plasma-Lyte A (Baxter, Deerfield, IL, USA) fluids was used.

At the conclusion of surgery, the arthroscopic portals were closed with a 2-0 polypropylene suture in a simple interrupted pattern, 1 g of amikacin was administered intra-articularly, and an additional 1 g was delivered via regional limb perfusion using the cephalic vein. Regional limb was performed with an Esmarch tourniquet in place for 20 min. The surgical site was bandaged, and the horse recovered uneventfully from sedation.

**Figure 4:**
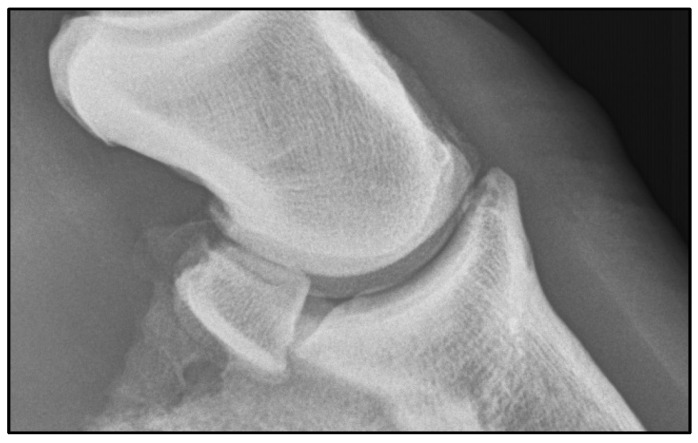
One-year post-operative lateral radiograph of the right fore distal interphalangeal joint.

The gelding was discharged the following day with no lameness at the walk. Post-operative medications included doxycycline (10 mg/kg PO, Q12 h) for 7 days and phenylbutazone at a dose of 1 g (PO, Q12 h) for 3 days, then 1 g (PO, Q24 h) for 3 days. Sutures were removed 2 weeks post-surgery, and the horse began hand walking and was allowed to undertake monitored turnout in a paddock of 24 feet by 24 feet or less. At 31 days post-surgery, 15 million autologous mesenchymal stem cells were injected into the distal interphalangeal joint. Under-saddle walking activity was started at 6 weeks following surgery. At 90 days following surgery, the horse underwent a veterinary examination and was noted to be sound at a trot on a straight line. Autologous protein solution was prepared and administered aseptically into the distal interphalangeal joint and the horse returned to large paddock turnout. On veterinary examination 5 months following surgery, the horse was sound in a straight line at the trot with mild lameness noted in the right forelimb only on sharp turns to the right. The horse returned to competition approximately 5 months following surgery and has remained at the expected level of performance for 1 year following surgery. Veterinary examination at 1 year post-surgery confirmed continued soundness at the trot; radiographic examination confirmed bone remodeling at the surgical site (Figure 4).

## 3. Discussion

Arthroscopy of the equine distal interphalangeal joint is a well-established surgical intervention, particularly for treatment of extensor process fragmentation, and is associated with positive outcomes when performed under general anesthesia. In a retrospective study of 18 Friesian horses, large extensor process fragments were successfully removed in all cases using arthroscopy via standard approach, in dorsal recumbency [9]. Furthermore, 14 of 17 of these horses returned to their intended use. Arthroscopic debridement of extensor process fragments under general anesthesia results in a good outcome with short-term resolution of lameness reported to be 85%, and 46% of horses remaining in full work 4 years following surgery [10].

In contrast to the traditional approach using a rigid arthroscope, nanoneedle arthroscopy may present a minimally invasive alternative that is feasible in standing, sedated horses, mitigating the risks of general anesthesia while still accomplishing therapeutic goals. The current published evidence supporting standing therapeutic needle arthroscopy has focused on fetlock joints, where successful removal of dorsal osteochondral fragments has been reported with minimal complications [8]. This case report intends to extend the applications of standing therapeutic needle arthroscopy to the distal interphalangeal joint. Use of the Arthrex NanoNeedle arthroscope may provide several technical advantages that facilitate successful standing intervention. The 10° field of view of the NanoNeedle scope, in combination with the 3.3 mm outer diameter high-flow cannula, provided excellent visualization of the extensor process of the third phalanx and the distal condyles of the second phalanx in this case. The coffin joint space and cartilage characteristics are altered by positioning in weight-bearing compared to non-weight-bearing conditions. A comparative low-field MRI study found the cartilage to be significantly thinner under load, representative of the compressive deformation occurring during normal gait [11]. The change in joint mechanics when weight-bearing suggests that the articular surface may be more accurately assessed in this natural position, allowing for detection of more subtle irregularities during surgery.

Maintenance of joint distension with the high-flow cannula and an arthroscopic pump provided consistent visualization and instrument manipulation throughout the procedure. The overall maneuverability of instrumentation and joint visualization in the weight-bearing position was subjectively superior to that typically encountered during arthroscopy performed under general anesthesia. The biomechanics of the equine distal interphalangeal joint have largely been explored in relation to soft tissue strain and compensation for the hoof capsule; however, the flexion and extension profile is associated with the stance phase of the gait, where extension is appreciated as the weight is transferred onto the grounded hoof [12]. This suggests that despite forced extension under general anesthesia, loading of the joint may remain superior for intra-articular maneuverability.

In this case, the increased maneuverability afforded by the 2.2 mm arthroscope, along with the shorter length and lightweight profile of the NanoNeedle scope and Nano Oval Burr, was particularly advantageous in the confined space of the coffin joint. Portal size was minimized compared with rigid arthroscopes during initial exploration, and no iatrogenic cartilage damage was appreciated. Precise instrument control is critical in standing arthroscopy as patient movement presents an inherent challenge and risk for cartilage injury. The small instrument profile and improved maneuverability appreciated in our case is in alignment with prior descriptions of needle arthroscopy benefits, particularly when used in anatomically constrained regions [8].

Similar to previous reports of standing needle arthroscopy in the fetlock, minor intraoperative hemarthrosis was encountered but was readily managed and did not adversely affect outcome, further supporting the safety of this approach in appropriately selected cases [8]. Ergonomic challenges associated with the standing setup, including fluid management and limited draping, required careful operating room organization consistent with previous discussion [13]. Given the proximity of the portals to the ground, meticulous positioning is essential to maintain sterility. Refinement of these procedural elements is expected to improve surgical efficiency and further enhance the practicality of standing NanoNeedle arthroscopy of the distal interphalangeal joint.

Standing arthroscopy has several important limitations that warrant consideration. Not all joints are readily accessible in the standing horse, and the ability to manipulate the limb during the procedure is inherently restricted. In addition, even with appropriate restraint, patient movement may occur, resulting in a less stable and less consistent surgical field. Consequently, standing arthroscopy may be considered more technically demanding than procedures performed under general anesthesia. Given these constraints, standing arthroscopy is generally limited to diagnostic applications and relatively minor surgical interventions, such as debridement or fragment removal. Successful execution of standing arthroscopy depends on the use of well-designed and executed sedation protocols that provide sufficient analgesia to minimize patient movement and ensure the safety of both the patient and surgical personnel.

## 4. Conclusions

This technique provided both diagnostic and therapeutic access to the fore DIP joint using the NanoNeedle scope in the standing horse, demonstrating the feasibility of this approach. The use of the NanoNeedle scope presents a procedural advantage to the rigid arthroscope in the standing horse, with improved maneuverability and surgical ergonomics and reduced iatrogenic injury.

## Data Availability

Due the confidentiality of the animal’s medical records, specific information may be available upon request.
